# The impact of variation in out-of-hours doctors’ referral practices: a Norwegian registry-based observational study

**DOI:** 10.1093/fampra/cmad014

**Published:** 2023-02-18

**Authors:** Jesper Blinkenberg, Øystein Hetlevik, Hogne Sandvik, Valborg Baste, Steinar Hunskaar

**Affiliations:** National Centre for Emergency Primary Health Care, NORCE Norwegian Research Centre AS, Årstadveien 17, 5009 Bergen, Norway; Department of Global Public Health and Primary Care, University of Bergen, Årstadveien 17, 5009 Bergen, Norway; Department of Global Public Health and Primary Care, University of Bergen, Årstadveien 17, 5009 Bergen, Norway; National Centre for Emergency Primary Health Care, NORCE Norwegian Research Centre AS, Årstadveien 17, 5009 Bergen, Norway; National Centre for Emergency Primary Health Care, NORCE Norwegian Research Centre AS, Årstadveien 17, 5009 Bergen, Norway; National Centre for Emergency Primary Health Care, NORCE Norwegian Research Centre AS, Årstadveien 17, 5009 Bergen, Norway; Department of Global Public Health and Primary Care, University of Bergen, Årstadveien 17, 5009 Bergen, Norway

**Keywords:** emergencies, gatekeeping, general practitioners, out-of-hours medical care, patient admission, referral and consultation

## Abstract

**Background:**

In a gatekeeping system, the individual doctor’s referral practice is an important factor for hospital activity and patient safety.

**Objective:**

The aim of the study was to investigate the variation in out-of-hours (OOH) doctors’ referral practice, and to explore these variations’ impact on admissions for selected diagnoses reflecting severity, and 30-day mortality.

**Methods:**

National data from the doctors’ claims database were linked with hospital data in the Norwegian Patient Registry. Based on the doctor’s individual referral rate adjusted for local organizational factors, the doctors were sorted into quartiles of low-, medium-low-, medium-high-, and high-referral practice. The relative risk (RR) for all referrals and for selected discharge diagnoses was calculated using generalized linear models.

**Results:**

The OOH doctors’ mean referral rate was 110 referrals per 1,000 consultations. Patients seeing a doctor in the highest referring practice quartile had higher likelihood of being referred to hospital and diagnosed with the symptom of pain in throat and chest, abdominal pain, and dizziness compared with the medium-low quartile (RR 1.63, 1.49, and 1.95). For the critical conditions of acute myocardial infarction, acute appendicitis, pulmonary embolism, and stroke, we found a similar, but weaker, association (RR 1.38, 1.32, 1.24, and 1.19). The 30-day mortality among patients not referred did not differ between the quartiles.

**Conclusions:**

Doctors with high-referral practice referred more patients who were later discharged with all types of diagnoses, including serious and critical conditions. With low-referral practice, severe conditions might have been overlooked, although the 30-day mortality was not affected.

**Key messages**

- There is a considerable variation in referral practice between OOH doctors.
- OOH doctors with high-referral practice refer more patients with minor conditions.
- OOH doctors with low-referral practice probably overlook some critical conditions.
- There is no difference in 30-day mortality between the not-referred patients.

## Background

Primary care doctors perform gatekeeping for acute referrals to hospital in many healthcare systems.^1,2^ Outside opening hours, such gatekeeping is performed by doctors in out-of-hours (OOH) services.^3–7^ Gatekeeping systems have shown to reduce hospital admissions.^1,2^ However, there is an ongoing debate on the impact of the primary care gatekeeping role regarding healthcare utilization and patient safety.^2,8^

Previous studies have shown considerable variation in referral rates between OOH doctors in England, resulting in a discussion of whether introducing measures to reduce the referral rates to decrease hospital workloads.^4,9,10^ In Norway, variation in general practitioners’ (GPs’) admission practice when working OOH has led to a discussion about patient safety for patients meeting low-referring doctors.^3^ Gatekeeping acute referrals to hospital captures a major dilemma, not missing severe or critically ill patients without overloading hospital capacity.^11^

Models of diagnostic reasoning in primary care have described elements like medical decision making, medical problem solving, and doctor’s gut feeling.^12,13^ These may be affected by the clinician’s perception of risk, thus leading to different referral practices in acute cases.^14^ The effect of different degrees of gatekeeping on healthcare management and patient safety has been explored.^3,15^ Still, this is not fully understood in respect to critical conditions and avoidable emergency admissions.

In this study, we wanted to investigate variation in the individual OOH doctors’ referral practices and to identify doctor characteristics associated with high- or low-referral practice. For some selected hospital diagnoses illustrating different levels of severity, we wanted to explore how referral practices impact the admissions. Further, we wanted to analyse the association between referral practice and 30-day mortality.

## Methods

The Norwegian health care system comprises a strong primary healthcare system. Municipalities are responsible for primary health care, including regular GPs and OOH services.^5,6^ OOH services provide acute care outside the opening hours of the regular GPs’ surgeries and daytime when the regular GP is not available. GPs and interns (mandatory first 18 months of specialization) are obliged to perform OOH services. The state organizes the ambulance services and hospitals.

### Data sources

The study was based on registry data from national health registries covering the whole population in Norway.

The Control and Payment of Reimbursement to Health Service Providers database (KUHR) receives claims from all general practice and OOH service contacts. Every claim includes information about the patients national identification number, sex, and age of the patient, time and date for the contact, the type of contact (telephone contact, consultation, or home visit), a diagnosis according to the International Classification of Primary Care, second edition (ICPC-2), whether the contact was from a GP practice or an OOH service, the practice municipality, the identification number of the doctor, and if the doctor is an approved specialist in general practice or not.

The Norwegian Patient Registry (NPR) contains information on all somatic hospital stays and includes information on the patient’s national identification number, time and date of the admission, duration of stay, degree of urgency, and 1 or more discharge diagnoses according to International Statistical Classification of Diseases and Related Health Problems version 10 (ICD-10).

Statistics Norway replaced the national identification numbers in the KUHR and NPR databases with pseudo-anonymized identification numbers. Thus, data from KUHR and NPR and information on deaths from Statistics Norway could be combined without revealing the patients’ identities.

### Study population, variables, and definitions

#### OOH consultation

**Fig. 1.**
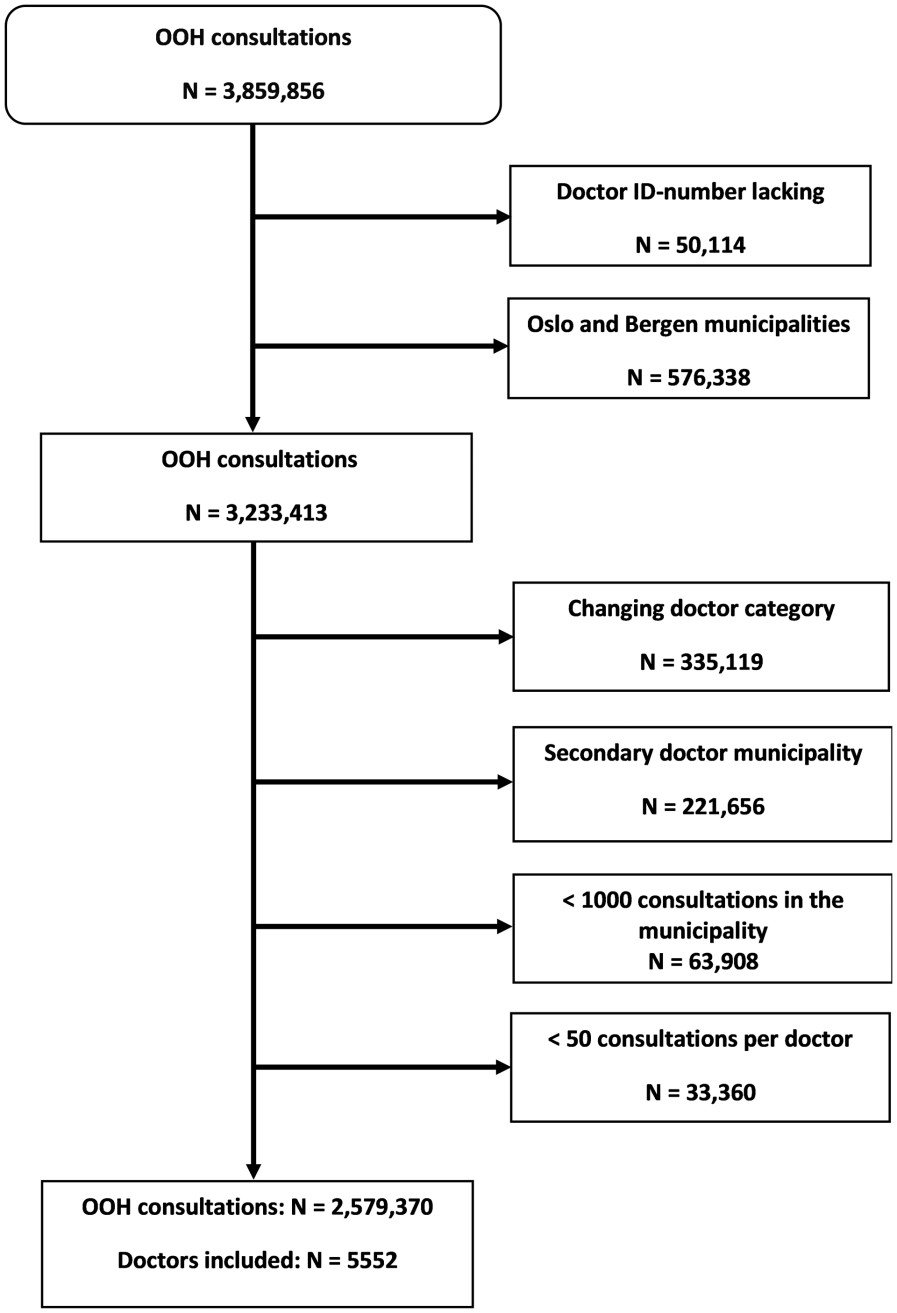
Consultations were excluded for doctors lacking identification number and consultation originating from a municipality excluded due to low OOH activity or divergent OOH clinics. If the doctor changed doctor category (intern, GP, or OOH physician) during the study period only the consultations as the first category were included. For doctors working in different municipalities, we kept the consultations from the municipality with the highest number of consultations.

In this study, we defined an OOH consultation as an OOH contact in the KUHR registry with a consultation code during the years 2016, 2017, or 2018. Claims missing the doctor identification number were excluded (Fig. 1). We also excluded all data from the 2 largest municipalities (Oslo and Bergen) because of organizational factors. Local organization models with several clinics in each of these 2 municipalities with different patient selections gave divergent referral practices within the municipalities resulting in clusters of referral practice, and data from the municipalities were therefore inappropriate for our analyses.

#### Acute referral

NPR provided data on all acute hospital admissions. An admission was included if it could be linked to an OOH consultation within 24 h before the admission. Psychiatric hospital admissions were not included. We used the 3-character main ICD-10 diagnosis from the hospital as the discharge diagnosis. Hospital stay was given as completed days.

#### Doctor categories

For each year (2016, 2017, and 2018) all doctors were sorted into 3 categories: Interns (intern consultations in the same year), GPs (GP consultations but no consultations as an intern in the same year), or OOH physicians (no intern or GP consultations in the same year). If the doctor changed category during the study period 2016–2018 we kept the consultations related to the first category for that doctor and later consultations were excluded (Fig. 1). Likewise, for doctors working in different municipalities we kept the one with the highest number of consultations. OOH consultations performed in municipalities with fewer than 1,000 consultations during 2016–2018 were excluded.

All remaining doctors were then sorted according to their OOH activity as <150, 150–399, 400–799, and ≥800 consultations during the study period. Doctors with fewer than 50 consultations were excluded.

#### Patient morbidity

To adjust for patient morbidity in the primary care setting, diagnoses in all contacts from KUHR from 2013 to 2015 were used to calculate the patients’ morbidity load using a newly developed ICPC-2 morbidity index.^16^

#### Referral rates and referring practices

The individual doctor’s referral rate was calculated by dividing the number of acute hospital referrals by all consultations by that doctor during the study period. To reduce confounding and take into account possible variation in referral rates from different municipalities and OOH services (due to access, geography, distance, or patient selection) the individual doctor’s referral rate was adjusted based on the total referral rate for each municipality. This was done by dividing the doctor’s referral rate by the referral rate for the pertaining municipality. This ratio was then used to define each doctor’s referring practice (relative to the other doctors in the same municipality) and to group them in quartiles (low-, medium-low-, medium-high-, and high-referral practice).

To assess the referral practice’s impact on admissions with some selected important conditions, we calculated a ratio for 8 different discharge diagnoses. The total number of each of the selected discharge diagnoses in each referral practice quartile were divided by the total number of patient consultations in the same quartile. The selected critical conditions were conditions where acute hospital admission is standard procedure in acute care. We also analysed selected corresponding symptom-describing diagnoses. The ICD-10 diagnoses used were pain in throat and chest (R07), acute myocardial infarction (AMI) (I21), abdominal pain (R10), acute appendicitis (K35), abnormal breathing (R06), pulmonary embolism (I26), dizziness (R42), and cerebral infarction (I63).

### Statistical analyses

Distributions of doctor characteristics (sex, age, type of doctor, GP specialist status, and OOH activity) were described in frequency tables including the mean referral rate and by the distribution in the referral practice quartiles. The relative risk (RR) of being in the high- versus the low-referral practice quartile was estimated for doctors’ age (30–39 years as the reference), for female doctors, for doctor category (GP as the reference), for GP speciality status, and for OOH activity (≥800 consultations as the reference). We used 3 generalized linear models (log-binomial), crude, adjusted for the other doctors’ factors, and adjusted for patient factors in addition to the other doctors’ factors. Patient factors that were adjusted for included age (<16 or >69 years), sex, morbidity (ICPC-2 morbidity index value 0 versus value 1–3), and night consultations versus day or evening consultations.

The RR of being referred to an acute hospital admission after an OOH consultation was calculated for the 4 doctor referral practice quartiles. The doctors in the medium-low quartile had the highest numbers of consultations and were used as the reference group in the analyses. Further, the RR for a referral to hospital and receiving a specific ICD-10 diagnosis given at hospital after an OOH consultation was calculated for the doctor referral practice quartiles. We used generalized linear models to estimate the RR with 95% confidence intervals (CIs) for both the crude model and the model adjusted for patient characteristics (age, sex, morbidity, and night consultations versus day or evening consultations). Corresponding analyses were performed to estimate the RR of death within 30 days for referred and not-referred patients.

The analyses were performed using Stata 16.1. (StataCorp. 2019. Stata Statistical Software: Release 16. College Station, TX: StataCorp LLC.)

## Results

We identified 5,552 doctors with 2,579,370 consultations leading to 259,648 acute referrals to hospital (Fig. 1). The doctors’ mean crude referral rate was 110 referrals per 1,000 consultations (Table 1). Younger doctors, female doctors, and doctors with few OOH consultations had higher mean referral rates (Table 1). Referral rates by patient factors are shown in the Supplementary Material.

**Table 1.** OOH doctor characteristics and referral rates for acute hospital referrals from Norway 2016–2018, including quartile distributions for doctors by their referral practice.

Doctor characteristics				Referral practice^a^			
	All			Low	Medium-low	Medium-high	High
	*N*	%	Mean referral rate/1,000 consultations	%	%	%	%
All	5,552		110				
Sex							
Female	2,527	46	117	37	39	47	59
Male	3,024	54	103	63	61	53	41
Age (years)							
<30	1,352	24	118	20	22	25	31
30–39	2,459	44	114	38	45	46	48
40–49	1,058	19	104	23	20	19	15
50–59	492	9	94	12	10	8	5
≥60	191	3	82	7	3	2	2
Doctor category							
GPs	3,055	55	106	62	60	55	43
Interns	1,901	34	119	27	30	33	47
OOH physicians	596	11	110	11	9	12	11
GP specialist							
Yes	1,413	23	103	29	27	23	15
No	4,687	77	112	71	73	77	85
OOH activity (consultations)							
<150	2,138	39	118	38	30	33	53
150–399	1,797	32	109	31	33	35	31
400–799	898	16	101	17	20	17	10
≥800	719	13	96	14	18	15	5

^a^The doctor’s referral practice was calculated relative to the referral rate of their municipality.

**Fig. 2.**
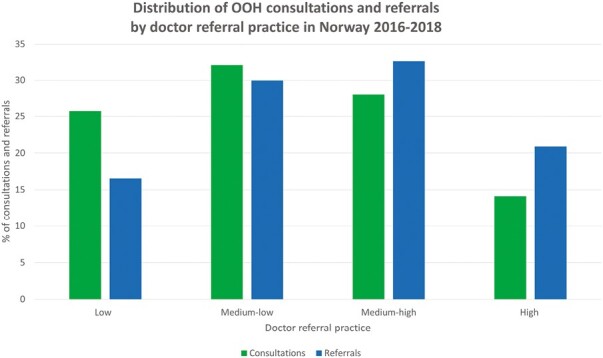
Distribution of 2,579,370 OOH consultations leading to 259,648 acute referrals to hospital. Consultations and referrals performed by 5,552 OOH doctors sorted into referral practice quartiles (low, medium-low, medium-high, and high).

Each doctor referral practice quartile (low to high) contained 1,388 doctors. The doctors in the low-referral practice quartile had 26% of the consultations but accounted for only 17% of the referrals (Fig. 2). The doctors in the high-referral practice quartile had only 14% of the consultations but accounted for 21% of the referrals. The likelihood for a doctor to be in the high- versus the low-referral practice quartile varied with doctor factors, also when adjusted for other doctor factors and patient factors (Table 2).

**Table 2.** Doctor and patient factors as explanatory variables for OOH doctors’ risk of being in the high- versus the low-referral practice quartile in Norway 2016–2018.

Doctor factors	High-referral practice quartile					
			Adjusted for			
	Crude		Doctor factors		Doctor and patient factors^a^	
	RR	95% CI	RR	95% CI	RR	95% CI
Age						
<30	1.09	1.01–1.18	0.93	0.86–1.02	0.96	0.89–1.04
30–39	Ref.		Ref.		Ref.	
40–49	0.71	0.63–0.80	0.83	0.74–0.96	0.84	0.74–0.95
50–59	0.51	0.41–0.63	0.64	0.52–0.80	0.65	0.53–0.80
≥60	0.36	0.25–0.51	0.47	0.32–067	0.47	0.33–0.67
Sex						
Male	Ref.		Ref.		Ref.	
Female	1.54	1.43–1.67	1.34	1.24–1.44	1.33	1.24–1.43
Doctor category						
GP	Ref.		Ref.		Ref.	
Intern	1.56	1.44–1.69	1.23	1.12–1.35	1.23	1.13–1.35
OOH physician	1.19	1.04–1.35	1.27	1.11–1.45	1.19	1.04–1.36
Specialty						
No	Ref.		Ref.		Ref.	
Yes	0.63	0.57–0.71	0.94	0.82–1.08	0.94	0.82–1.08
OOH activity						
≥800	Ref.		Ref.		Ref.	
400–799	1.39	1.10–1.76	1.26	1.00–1.58	1.22	0.99–1.50
150–399	1.86	1.51–2.29	1.57	1.28–1.92	1.51	1.25–1.82
<150	2.16	1.76–2.64	1.64	1.34–2.01	1.48	1.22–1.79

^a^Patient factors included in the analysis: age (<16 or >69 years), sex, morbidity, or a consultation at night.

The mean referral rates in the referral practice quartiles (low, medium-low, medium-high, and high) were 65, 94, 117, and 149 referrals per 1,000 consultations, respectively (Table 3). As a consequence of the definition of the quartiles, a patient consulting a doctor in the highest referral practice quartile had a higher RR for referral to hospital compared with a patient from the medium-low-referral practice quartile (RR 1.46) (Table 3). However, for patients consulting a doctor from the highest referring practice quartile, the risk to be referred to hospital and then discharged with a symptom ICD-10 diagnosis was even higher. For the diagnoses of chest pain (R07), abdominal pain (R10), abnormal breathing (R06), and dizziness (R42), the adjusted RRs were 1.63, 1.49, 1.43, and 1.95, respectively, relative to the medium-low quartile (Table 3). Also, the risk of being diagnosed with a critical condition after an OOH consultation increased with increasing referral practice. The RR to be referred and discharged with AMI (I21), acute appendicitis (K35), pulmonary embolism (I26), or cerebral infarction (I63) if the attending a doctor was in the high-referral practice quartile compared with the medium-low quartile was 1.38, 1.32, 1.24, and 1.19, respectively. The frequency of hospital stays <24 h increased from the low- to the high-referral practice quartile (10–17%) (Table 3).

**Table 3.** Acute referrals from OOH doctors according to their referral practice (low, medium-low, medium-high, and high referral practice) and percentage of referrals where the patient was discharged within 24 h in Norway 2016–2018, including RR for referral after a consultation according to doctors’ referral practice and for different hospital diagnoses, adjusted for patient factors (age, sex, morbidity, and night consultation).

	Referrals		Hospital stay <24 h (%)	RR^b^			
	*N*	‰^a^		Crude	95% CI	Adjusted	95% CI
All consultations (*N*)							
Low quartile (663,402)	42,935	64.7	10.0	0.69	0.66–0.72	0.71	0.68–0.75
Medium-low (827,509)	77,775	94.0	11.2	Ref.		Ref.	
Medium-high (724,028)	84,649	116.9	13.4	1.24	1.18–1.31	1.22	1.15–1.28
High quartile (364,431)	54,289	149.0	17.0	1.59	1.52–1.66	1.46	1.40–1.53
Hospital diagnoses (ICD-10)							
Pain in throat and chest (R07)							
Low quartile	1,284	1.9	23.1	0.57	0.50–0.64	0.60	0.53–0.68
Medium-low	2,819	3.4	25.3	Ref.		Ref.	
Medium-high	3,419	4.7	31.0	1.39	1.24–1.54	1.33	1.19–1.48
High quartile	2,295	6.3	37.2	1.85	1.67–2.05	1.63	1.47–1.81
Acute myocardial infarction (I21)							
Low quartile	828	1.2	9.9	0.73	0.66–0.81	0.79	0.71–0.87
Medium-low	1,408	1.7	10.8	Ref.		Ref.	
Medium-high	1,484	2.0	8.5	1.20	1.09–1.33	1.17	1.07–1.28
High quartile	967	2.7	11.9	1.56	1.41–1.72	1.38	1.26–1.52
Abdominal pain (R10)							
Low quartile	1,758	2.6	18.7	0.63	0.57–0.69	0.66	0.60–0.72
Medium-low	3,500	4.2	21.9	Ref.		Ref.	
Medium-high	4,036	5.6	24.9	1.32	1.18–1.48	1.24	1.10–1.39
High quartile	2,605	7.1	30.5	1.69	1.54–1.85	1.49	1.36–1.63
Acute appendicitis (K35)							
Low quartile	1,059	1.6	1.4	0.75	0.69–0.82	0.78	0.71–0.85
Medium-low	1,750	2.1	2.3	Ref.		Ref.	
Medium-high	1,859	2.6	2.2	1.21	1.12–1.32	1.16	1.00–1.26
High quartile	1,102	3.0	2.5	1.43	1.31–1.56	1.32	1.21–1.43
Abnormal breathing (R06)							
Low quartile	107	0.2	17.8	0.50	0.39–0.64	0.52	0.41–0.67
Medium-low	265	0.3	16.6	Ref.		Ref.	
Medium-high	297	0.4	27.3	1.28	1.07–1.54	1.25	1.04–1.50
High quartile	182	0.5	27.5	1.56	1.27–1.91	1.43	1.17–1.75
Pulmonary embolism (I26)							
Low quartile	206	0.3	1.5	0.64	0.53–0.77	0.67	0.56–0.81
Medium-low	403	0.5	2.2	Ref.		Ref.	
Medium-high	434	0.6	4.4	1.23	1.05–1.44	1.21	1.03–1.41
High quartile	243	0.7	3.7	1.37	1.16–1.62	1.24	1.05–1.47
Dizziness (R42)							
Low quartile	290	0.4	11.3	0.63	0.53–0.74	0.65	0.55–0.77
Medium-low	575	0.7	7.7	Ref.		Ref.	
Medium-high	801	1.1	10.4	1.59	1.39–1.82	1.61	1.40–1.84
High quartile	512	1.4	14.7	2.02	1.74–2.35	1.95	1.67–2.27
Cerebral infarction (I63)							
Low quartile	524	0.8	1.5	0.67	0.60–0.76	0.70	0.62–0.79
Medium-low	969	1.2	0.9	Ref.		Ref.	
Medium-high	878	1.2	0.8	1.04	0.93–1.15	1.06	0.95–1.18
High quartile	522	1.4	1.0	1.22	1.09–1.38	1.19	1.06–1.33

^a^Of all consultations with OOH doctor in the same quartile.

^b^RR for hospital stay in total and related to different diagnoses given in hospital.

The 30-day mortality was lower for patients referred to hospital in the medium-high- and high-referral practice quartiles compared with the medium-low quartile (RR 0.91 and 0.90, respectively). However, there was no difference in the 30-day mortality between the quartiles for patients not referred to hospital (Supplementary Material).

## Discussion

### Main results

The mean referral rate for OOH doctors was 110 referrals per 1,000 consultations. The doctors in the referral practice quartiles (low, medium-low, medium-high, and high) had mean referral rates of 65, 94, 117, and 149 referrals per 1,000 consultations, respectively. Female doctor, young doctors, interns, and doctors with low OOH activity were more likely to be in the high-referral practice quartile. In the high-referring practice quartile, there was a higher likelihood for the patients to be referred to hospital and diagnosed with the ICD-10 symptom diagnoses of pain in throat and chest, abdominal pain, abnormal breathing, and dizziness. However, we also found an increasing risk from low- to high-referral practice for a patient seen at OOH services to be discharged from hospital with potentially severe conditions like AMI, acute appendicitis, pulmonary embolism, and stroke. The 30-day mortality was lower for referred patients who had been attending a doctor with higher referral practice, but there was no difference in 30-day mortality for the not-referred patients.

### Strengths and limitations

This study included national data on OOH activity and acute referrals to hospital in Norway in 2016–2018. This ensured sufficient numbers of consultations, referrals, and doctors to explore the doctors’ referral rate.

The OOH consultations in the KUHR registry were linked to the acute hospital admissions in the NPR registry if the admission occurred within 24 h after the consultation. This linkage has some uncertainties. The OOH consultation might have been random and not related to the admission, but previous studies have shown an accumulation of primary care consultations 24 h before admission and a strong relationship between the primary care diagnoses and the discharge diagnoses given at hospital.^5,6,11^ This supports the design of the present study.

There is a substantial variation in Norwegian OOH services when it comes to local organizational factors such as telephone triage routines, OOH accessibility, and prehospital ambulance procedures for critical conditions. This affects the doctors’ referral rate. Therefore, the doctors’ individual referral rate was adjusted to the overall referral rate in the municipality. The doctors were compared only to doctors working under the same conditions in the same municipality when their referral practice was calculated, and this eliminated confounding due to local organizational factors affecting referral practice.

The information on diagnostic codes did not contain information on the severity of the condition, e.g. a minor versus a severe myocardial infarction, uncomplicated appendicitis or a perforated appendix. The length of hospital stay might indicate severity, but some conditions have treatment protocols resulting in homogenous durations of hospital stays that do not capture the severity gradient. We have not taken into consideration that patients with critical conditions who are not referred might recontact health services and have a delayed referral.

### Referral practice

As expected, we found that younger and female doctors had higher referral rates, and the association was even stronger for doctors with few OOH consultations. This fits well with previous studies,^3,4,9^ but the more than doubled mean referral rate between the low- and the high-referring quartile cannot be explained by simple doctor characteristics alone.^9^ Individual tolerance for risk and uncertainty is an important factor for explaining variations in referral practice.^14,17^ Doctors in the lower-referring practice quartiles might use “wait and see” as a clinical diagnostic tool, e.g. for abdominal pain, more actively compared with the high-referring practice quartile, as an expression of higher tolerance for risk, whereas doctors in the high-referral practice quartile will prefer to admit if in doubt.^14,17^

### Symptom diagnoses

Given a symptom diagnosis at hospital indicates that no specific disease is revealed. The risk to be referred and diagnosed with a symptom diagnosis at hospital, after the OOH consultation increased from the low-referral practice quartile to the higher-referral practices. This was expected and was the rationale behind the aim to reduce referral rates.^4,17^ Considering that the referred patients in the high- and medium-high-referral practice quartiles had lower 30-day mortality, this could be safe. To reduce referrals where no disease is revealed will save both patient concern and inconvenience, and hospital workload and costs.^1,2,4,9,15,17^

We found that patients referred from doctors with high-referring practice more often had short hospital stays (<24 h) when discharged with symptom diagnoses. This could be an indication of possible avoidable referrals where little health benefit is gained but healthcare costs increase. It may also indicate effectively performed necessary investigations to exclude critical conditions.

### Critical conditions

Both stroke and AMI are sometimes misdiagnosed even when presenting with typical symptoms.^18,19^ It is also well known that undergoing myocardial infarctions or a cerebral infarction may show no, or vague symptoms termed as silent or unrecognized heart infarction or stroke.^20,21^ Acute appendicitis is sometimes missed at the first assessment, but acute appendicitis is also known to be a self-limiting condition in many cases.^22,23^ The increasing prevalence of pulmonary embolism may be due to better diagnostic tools or to increased awareness of the conditions.^24^

The likelihood to be referred and diagnosed with a critical condition, AMI, acute appendicitis, pulmonary embolism, or cerebral infarction, was higher in the high-referral practice quartile. Although the effect was smaller than for the symptom-describing hospital diagnoses, this implies that some critical conditions are probably overlooked in patients meeting a doctor in the low-referring group. This is in line with previous findings for a group of critical conditions after OOH consultations with GPs, but this has not been shown for each critical diagnosis separately.^3^ The previous Norwegian study did not find any difference in 30-day mortality related to different referral practices, which was similar to our lack of difference in not-referred patients. Thus, we can assume that the possible missed cases of critical conditions might be less severe and with low risk of fatal outcome. In the acute phase of a critical condition the patient might present with vague or atypical symptoms and therefore not be referred by a doctor in the lower-referring quartile. Vague and atypical presentations are more common with the least severe cases. However, even if there were no difference in 30-day mortality, the overlooked cases of AMI, cerebral infarction, and pulmonary embolism probably affected both morbidity and long-term mortality. This warrants further research.

Our findings should be taken into consideration when implementing enhanced gatekeeping roles in OOH services in Europe.^7^ Strengthening the OOH framework for decision making regarding gatekeeping for acute hospital referrals should be emphasized rather than encouraging general strict gatekeeping.^3^ Also, higher referral rates should be accepted for less-experienced doctors.

## Conclusions

The referral rate decreased with doctor’s increased experience. High-referral practice may lead to more avoidable admissions, and low-referral practice may increase the risk for critical conditions being overlooked. Variation in individual referral rates has an impact on both hospital costs and patient safety and should be taken into account when planning the interface between primary care and hospitals. Strengthening prehospital decision making and diagnostics should be prioritized.

## Supplementary Material

cmad014_suppl_Supplementary_Material

cmad014_suppl_Supplementary_Checklist

cmad014_suppl_Supplementary_Podcast

## Data Availability

The data used in this study were delivered with restrictions only to be used under licence for researchers in the current study. These restrictions are given by The Norwegian Directorate of Health and Statistics Norway, and the data are therefore not publicly available. However, the registry data used in this study may be available from The Norwegian Directorate of Health and Statistics Norway after application to the Regional Ethical Committee and the Norwegian Data Protection Authority.

## Authors’ information

Data from the Norwegian Patient Registry been used in this publication. The interpretation and reporting of these data are the sole responsibility of the authors, and no endorsement by the Norwegian patient register is intended or should be inferred.
