# Supplementary material for: The impact of variation in out-of-hours doctors’ referral practices: a Norwegian registry-based observational study
Source: Fam Pract. 2023 Feb 18;40(5-6):728–36. doi: 10.1093/fampra/cmad014 (PMC10745277; doi:10.1093/fampra/cmad014)
Supplement: cmad014_suppl_Supplementary_Material [file cmad014_suppl_supplementary_material.pdf]

Supplementary material

Referral rates varied by patient factors. Male patients, high patient age, comorbidity, and consultation during the night were associated with higher referral rates. The highest referral rate was for patients older than 85 years (271 referrals per 1000 consultations) but varied much by doctor referral practice quartile (188–365 referrals per 1000 consultations). The lowest referral rate was for children 3–15 years (37 referrals per 1000 consultations), whereas the largest variation between low and high quartiles was found for children 0–2 years old (27–76 referrals per 1000 consultations).

Supplementary Table 1. Patient characteristics in out-of-hours consultations and acute referrals to hospital by consulting doctors' referral practice quartiles (low, medium-low, medium-high, high) in Norway 2016–2018.

Patient characteristics	OOH consultations (n = 2,579,370) %	Acute referrals to hospital (n = 259,648) %	Referral rate[2] (All)	Referral rate[2] by consulting doctor's referral practice			
				Low	Medium-low	Medium-high	High
All	100	100	101	65	94	117	149
Sex							
Female	53	50	95	60	88	111	144
Male	47	50	107	70	100	123	154
Age (years)							
0–2	7	3	45	27	41	53	76
3–15	16	6	37	24	34	42	58
16–29	21	13	62	38	56	73	93
30–49	22	18	83	52	76	98	126
50–69	19	26	136	89	127	160	197
70–84	11	23	211	140	200	246	292
≥85	4	11	271	188	259	307	365
Morbidity (ICPC-2)[1]							
0	80	65	81	51	75	95	124
1–3	20	35	180	121	172	207	245
Time							
Day	30	26	87	57	84	103	133
Evening	57	52	92	63	88	108	131
Night	13	21	169	113	157	173	212

[1] Morbidity was calculated by primary care diagnoses 2013–2015 in the International Classification of Primary Care, second edition.

[2] Referral rate shown as acute referrals to hospital by 1000 OOH consultations.

Supplementary Table 2. Relative risk (RR) for 30-days mortality after a consultation according to doctors' referral practice (low, medium-low, medium-high, and high) for patients not referred to hospital and for patients referred, adjusted for patient factors (age, sex, morbidity, and night consultation), Norway 2016–2018.

	RR			
	Crude	95% CI	Adjusted	95% CI
Patients not referred (n = 2,319,722)				
Low	1.02	0.89–1.16	1.04	0.92–1.16
Medium-low	Ref.		Ref.	
Medium-high	0.95	0.85–1.05	0.98	0.89–1.07
High	1.07	0.95–1.20	1.06	0.95–1.17
Patients referred (n = 259,648)				
Low	1.01	0.91–1.11	1.03	0.94–1.12
Medium-low	Ref.		Ref.	
Medium-high	0.86	0.78–0.94	0.91	0.84–0.98
High	0.84	0.76–0.92	0.90	0.82–0.98
